# Histone deacetylase 6 inhibition counteracts the epithelial–mesenchymal transition of peritoneal mesothelial cells and prevents peritoneal fibrosis

**DOI:** 10.18632/oncotarget.20982

**Published:** 2017-09-18

**Authors:** Liuqing Xu, Na Liu, Hongwei Gu, Hongrui Wang, Yingfeng Shi, Xiaoyan Ma, Shuchen Ma, Jun Ni, Min Tao, Andong Qiu, Shougang Zhuang

**Affiliations:** ^1^ Department of Nephrology, Shanghai East Hospital, Tongji University School of Medicine, Shanghai, China; ^2^ School of Life Sciences and Technology, Advanced Institute of Translational Medicine, Tongji University, Shanghai, China; ^3^ Department of Medicine, Rhode Island Hospital and Brown University School of Medicine, Providence, RI, USA

**Keywords:** peritoneal fibrosis, HDAC6, TGF-β signaling pathway, inflammation, vascular endothelial growth factor

## Abstract

The role of histone deacetylase 6 (HDAC6) in peritoneal fibrosis remains unknown. In this study, we examined the effect of HDAC6 inhibition on the epithelial–mesenchymal transition (EMT) of peritoneal mesothelial cells and development of peritoneal fibrosis. Treatment with tubastatin A, a highly selective HDAC6 inhibitor, or silencing of HDAC6 with siRNA inhibited transforming growth factor β1-induced EMT, as evidenced by decreased expression of α-smooth muscle actin, collagen I and preserved expression of E-cadherin in cultured human peritoneal mesothelial cells. In a mouse model of peritoneal fibrosis induced by high glucose dialysate, administration of TA prevented thickening of the submesothelial region and decreased expression of collagen I and α-SMA. Mechanistically, tubastatin A treatment inhibited expression of TGF-β1 and phosphorylation of Smad-3, epidermal growth factor receptor, STAT3, and NF-κBp65. HDAC6 inhibition also suppressed production of multiple inflammatory cytokines/chemokines and reduced the infiltration of macrophages to the injured peritoneum. Moreover, tubastatin A was effective in inhibiting peritoneal increase of CD31(+) blood vessels and expression of vascular endothelial growth factor in the injured peritoneum. Collectively, these results suggest that HDAC6 inhibition can attenuate peritoneal fibrosis by inhibiting multiple pro-fibrotic signaling pathways, EMT, inflammation and blood vessel formation.

## INTRODUCTION

Peritoneal dialysis (PD) is an effective alternative form of renal replacement therapy in patients with end-stage renal disease (ESRD) [1]. However, long-term exposure to a diversity of unfavorable factors including bio-incompatible PD solution, uremia, peritonitis and basic chronic kidney diseases [2], results in structural changes of the peritoneal membrane. These changes include loss of mesothelial cells, occurrence of epithelial-mesenchymal transition (EMT), thickening of the submesothelial region and induction of angiogenesis. As a result, peritoneal fibrosis develops and ultrafiltration declines, ultimately leading to PD failure. Therefore, it is important to seek effective strategies to prevent and treat peritoneal fibrosis [3, 4].

Peritoneal fibrosis is one of the major complications occurring in patients undergoing long-term peritoneal dialysis. It is characterized by an increased number of myofibroblasts and deposition of interstitial extracellular matrix [5]. Elucidation of the origin of myofibroblasts in the peritoneum remains an active field of research. Initially, most studies *in vitro* and *in vivo* supported the theory that injured mesothelial cells were the progenitor of myofibroblasts. However, recent two lineage tracing studies of mouse genetics results in some controversy regarding this issue [6]. Whereas Lua et al. demonstrated that approximately 17% of myofibroblasts are derived from mesothelial cells during peritoneal fibrosis [7], Chen et al. revealed that submesothelial fibroblasts are the major source of myofibroblast precursors [8]. Nevertheless, both studies still support the importance of mesothelial cells in the development of peritoneal fibrosis since injured mesothelial cells acquire a capability to produce multiple cytokines/growth factors that stimulate resident fibroblast activation and proliferation, thereby producing more ECM proteins and supporting the development of peritoneal fibrosis [7–9].

Epithelial-mesenchymal transition (EMT) is a process in which epithelial cells lose their characteristic cell-cell junctions and polarized cell-surface molecules while acquiring properties typical of mesenchymal cells. Many growth factors and cytokines are implicated in this process. Among them, transforming growth factor (TGF)-β1 has been documented to play a predominant role in the initiation and development of EMT as well as peritoneal fibrosis [1, 10–15]. TGF-β1 induces EMT and peritoneal fibrosis through activation of the Smad signaling pathway [1, 11, 13, 14, 16]. In addition, our recent studies have revealed that the activation of epidermal growth factor receptors (EGFR) is also involved in these processes [13].

Inflammation and angiogenesis also contribute to peritoneal fibrosis [4]. In response to peritoneal membrane injury, a variety of pro-inflammatory factors such as interleukin-1 (IL-1), interleukin-6 (IL-6), tumor necrosis factor-α (TNF-α) and monocyte chemoattractant protein-1 (MCP-1) are produced. In addition, leukocytes, especially macrophages, infiltrate the injured section where they produce more cytokines/chemokines [13]. Expression and production of these cytokines/chemokines are tightly regulated by transcriptional factors. NF-*κ*B and STAT3 are two important transcriptional factors that are activated and involved in the process of peritoneal fibrosis [17, 18]. In addition, peritoneal mesothelial cells (MCs), in particular transdifferentiated MCs, under various stimuli produce vascular endothelial growth factor (VEGF) [19–23], a potent growth factor promoting angiogenesis [13, 24].

It is well known that gene expression and the activation of signaling pathways is subjected to epigenetic regulation. Epigenetics is defined as the post-translational modifications that occur without any changes in the DNA sequence [25, 26]. Among several types of epigenetic modifications, histone acetylation has been most studied. Histone acetylation is positively regulated by histone acetyltransferases (HATs) and negatively regulated by histone deacetylases (HDACs). Currently, 18 HDACs have been identified in mammals and are classified into four groups: class I HDACs (HDAC1, 2, 3 and 8), class II HDACs, subdivided into class IIa (HDAC4, 5, 7 and 9) and IIb (HDAC6 and 10), class III HDACs (SIRT1-7) and class IV HDACs (HDAC11). Studies have shown that HDACs are associated with fibrosis in multiple organs, including kidneys, lungs and heart [27]. A recent report has also indicated that administration of suberoylanilide hydroxamicacid (SAHA), a class I HDAC inhibitor, attenuates peritoneal fibrosis [28], suggesting that peritoneal fibrosis is also subjected to regulation by HDACs. However, it remains unknown whether other classes or isoforms of HDACs are involved in the process of peritoneal fibrosis.

Among class II HDACs, HDAC6 is a unique cytoplasmic deacetylase that can deacetylate substrates such as α-tubulin, and regulate numerous biological processes, including cell migration, immune responses and the degradation of misfolded proteins [29, 30]. In contrast with other HDAC isoforms, HDAC6 KO mice do not demonstrate abnormal development or major organ dysfunctions [31], and some highly and potent selective inhibitors have also been identified for HDAC6. For example, tubastatin A (TA) has an IC50 for HDAC6 of 0.015 μM in a cell-free enzyme inhibition assay, representing more than a 1000-fold selectivity compared with HDAC isoforms 1–11 [32]. This provides a powerful tool to study its role in a variety of pathological conditions. Using this inhibitor, Choi et al., determined implications for HDAC6 in the development of kidney fibrosis and inflammation in a model of angiotensin II-infused mice [16]. In addition, we showed the importance of HDAC6 in mediating pathogenesis of acute kidney injury induced by rhabdomyolysis [16, 33].

Currently, the role of HDAC6 in peritoneal fibrosis remains unknown. Here, we examined its role in regulating the EMT of peritoneal mesothelial cells by utilizing TA and siRNA specifically targeting HDAC6. We also evaluated the efficacy of TA in preventing the development of peritoneal fibrosis in an animal model of chronic exposure to high glucose dialysate. Our results reveal a critical role of HDAC6 in mediating the EMT of mesothelial cells, activation of submesothelial fibroblasts and development of peritoneal fibrosis.

## RESULTS

### TA inhibits TGF-β1-induced EMT of cultured peritoneal mesothelial cells

**Figure 1:**
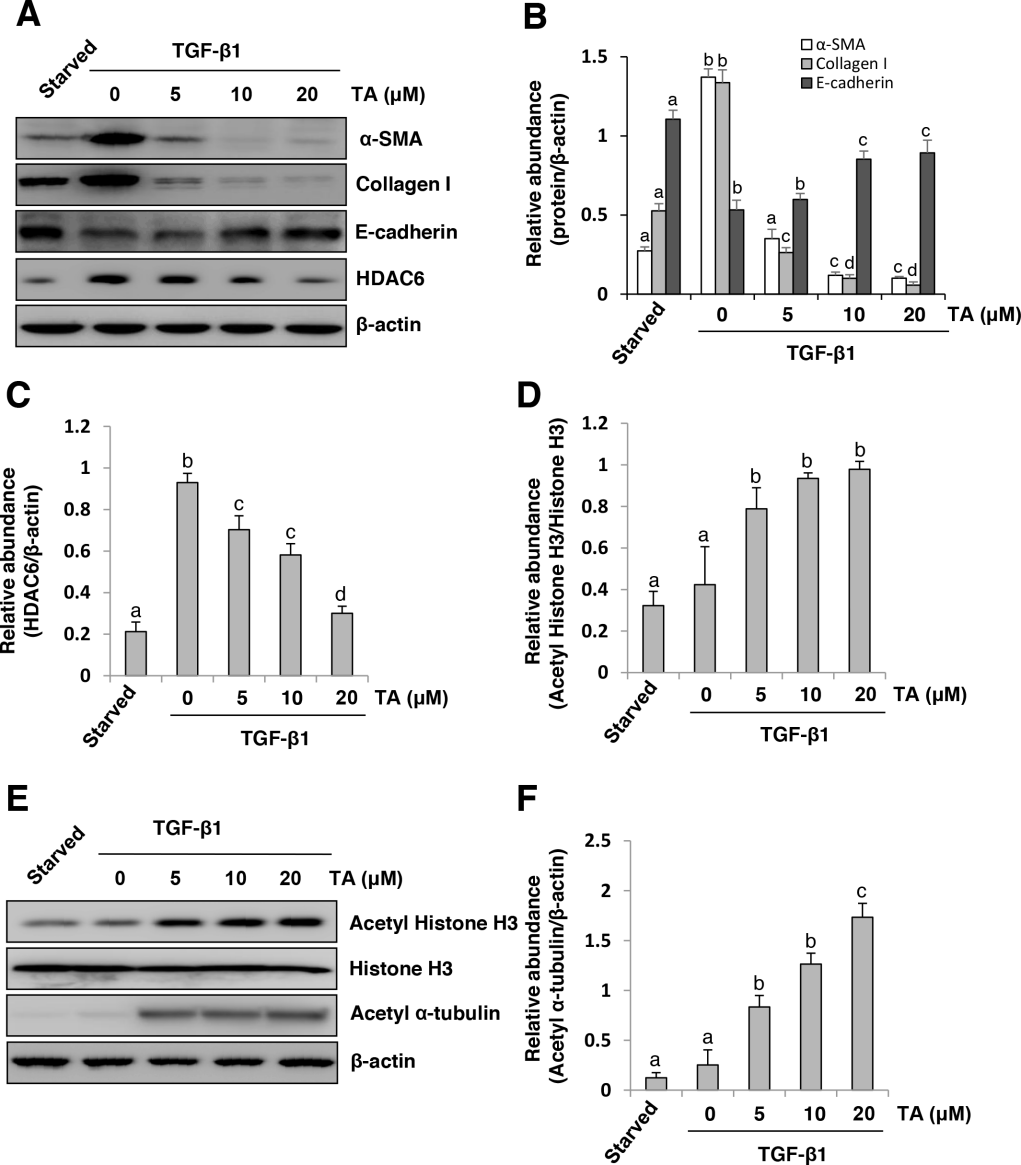
Inhibition of HDAC6 blocks TGF-β1-induced EMT of cultured human peritoneal mesothelia cells Serum-starved HPMCs were pretreated with various concentrations of TA (0-20 μM) for 1 hour and then exposed to TGF-β1 (10 ng/ml) for an additional 24h. **(A and E)** Cell lysates were subjected to immunoblot analysis with antibodies against various molecules as indicated. Expression levels of α-SMA, collagen I, E-cadherin **(B)** HDAC6 **(C)**, or acetyl α-tubulin **(F)** were quantified by densitometry and normalized with *β*-actin. **(D)** Expression level of acetyl histone H3 was quantified by densitometry and normalized with total histone H3. Values are means±SD of at least three independent experiments. Bars with different letters (a-d) for each molecule are significantly different from one another (*P*<0.05).

EMT of peritoneal mesothelial cells occurs after peritoneal damage and contributes to peritoneal fibrosis [9]. As a first step toward understanding whether HDAC6 is involved in the regulation of peritoneal fibrosis, we examined the effect of TA, a highly selective inhibitor of HDAC6 [34], on the EMT ofcultured human peritoneal mesothelial cells (HPMCs) in response to TGF-β1. Exposure of HPMCs to TGF-β1 at 10 ng/ml resulted in decreased expression of E-cadherin, a hallmark of epithelial cells and increased expression of α-SMA and collagen I, two hallmarks of EMT. In parallel, TGF-β1 induced an increase in the expression of HDAC6 and a slight decrease in the expression of acetyl-histone H3 and its specific target, acetyl α-tubulin. In contrast, treatment with TA inhibited TGF-β1-induced upregulation of α-SMA and collagen I and downregulation of E-cadherin, which occurred in a dose-dependent manner, with a maximum effect at 20 μM (Figure 1A, 1B). TA exposure also dose-dependently suppressed expression of HDAC6, which was accompanied by increased expression of acetyl-histone H3 and acetyl α-tubulin (Figure 1A, 1C, 1D-1F). Since histone H3 is a nuclear protein and α-tubulin is located in the cytosol, these data suggest that HDAC6 acts in both nucleus and cytosol to regulate EMT in peritoneal mesothelial cells.

### siRNA-mediated silencing of HDAC6 inhibits EMT of peritoneal mesothelial cells

**Figure 2:**
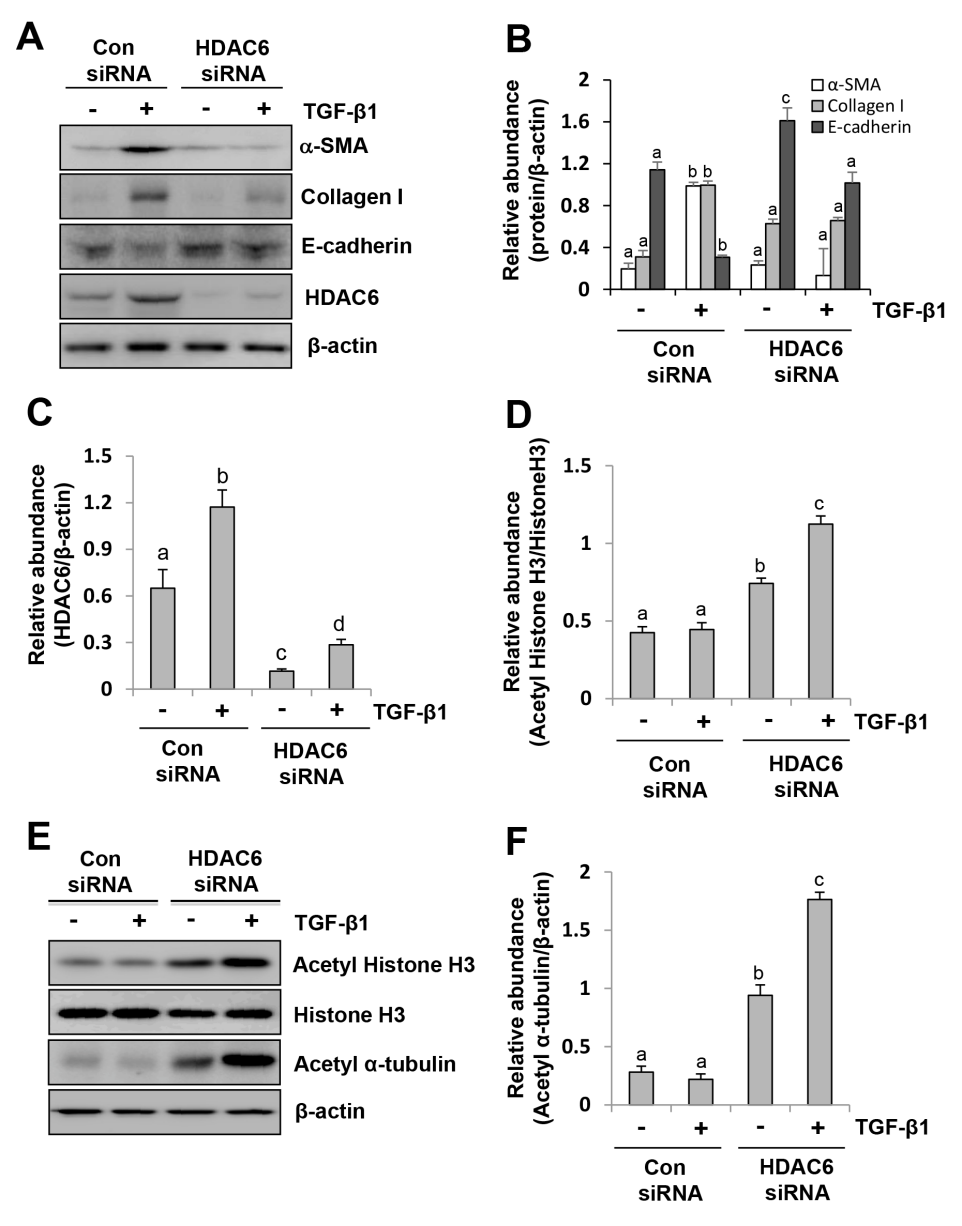
siRNA-mediated silencing of HDAC6 inhibits EMT of peritoneal mesothelial cells Serum-starved HPMCs were transferred with siRNA targeting HDAC6 or scrambled siRNA and then incubated with TGF-β1 (10 ng/ml) for an additional 24 hours. **(A and E)** Cell lysates were subjected to immunoblot analysis with antibodies against collagen I, α-SMA, E-cadherin, HDAC6, acetyl histone H3, acetyl α-tubulin, histone H3 or β-actin. Expression levels of collagen I, α-SMA, E-cadherin **(B)**, HDAC6 **(C)**, and acetyl α-tubulin **(F)** were quantified by densitometry and normalized with β-actin. Expression level of acetyl histone H3 was quantified by densitometry and normalized with histone H3 **(D)**. Values are mean±SD of at least three independent experiments. Bars with different letters (a-d) for each molecule are significantly different from one another (P<0.05).

To verify the inhibitory effect of TA on EMT, we examined the effect of HDAC6 knockdown on the EMT of peritoneal mesothelial cells using siRNA specifically targeting HDAC6. As shown in Figure 2A, reduction of HDAC6 expression by its specific siRNA also decreased TGF-β1 stimulated expression ofcollagen I and α-SMA and reciprocally increased expression of E-cadherin (Figure 2A–2C). As expected, downregulation of HDAC6 resulted in increased expression of acetyl histone H3 and acetyl α-tubulin without alteration of total H3 expression (Figure 2D–2F). These results further confirm the importance of HDAC6 in mediating EMT of peritoneal mesothelial cells.

### HDAC6 is required for activation of the TGF-β1/Smad3 signaling pathway in peritoneal mesothelial cells

**Figure 3:**
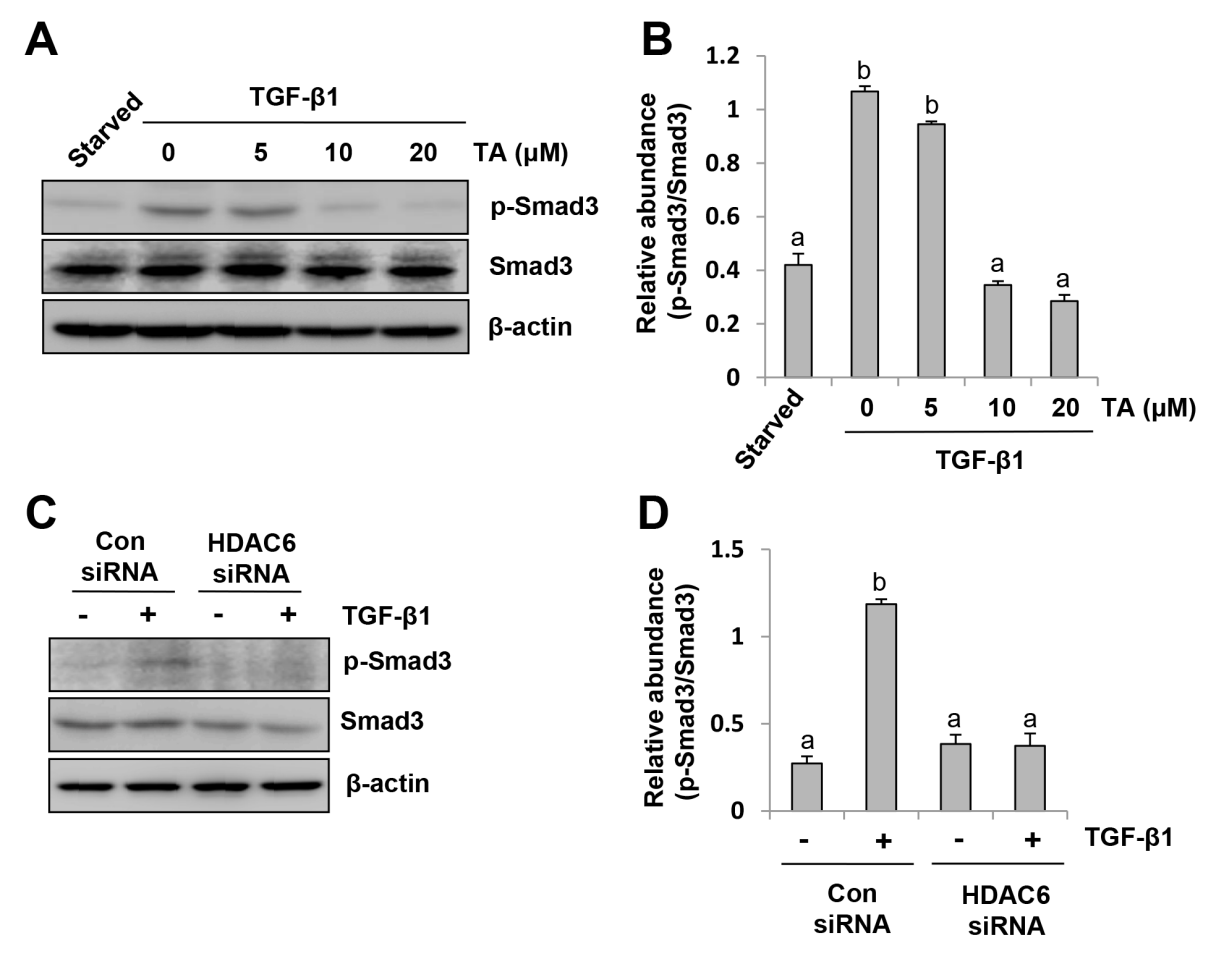
HDAC6 is required for TGF-β induced Smad3 phosphorylation in peritoneal mesothelial cells **(A)** Serum-starved HPMCs were pretreated with various concentrations of TA (0-20 μM) for 1 hour and then exposed to TGF-β1 (10 ng/ml) for an additional 24 hours. Cell lysates were subjected to immunoblot analysis with antibodies against p-Smad3, Smad3, or β-actin. **(B)** Expression level of p-Smad3 was quantified by densitometry and normalized with Smad3. **(C)** Serum-starved HPMCs were transferred with siRNA targeting HDAC6 or scrambled siRNA and then incubated with TGF-β1 (10 ng/ml) for an additional 24 hours. Cell lysates were subjected to immunoblot analysis with antibodies against p-Smad3, Smad3, or β-actin. **(D)** Expression level of p-Smad3 was quantified by densitometry and normalized with Smad3. Values are means±SD of at least three independent experiments. Bars with different letters (a-d) for each molecule are significantly different from one another (P<0.05).

It is well known that activation of the TGF-β1/Smad3 signaling is critical for the development of EMT in peritoneal mesothelial cells [9]. We thus examined whether HDAC6 would regulate EMT through activation of this signaling pathway in HPMCs. As shown in Figure 3A-3B, TA treatment dose-dependently suppressed Smad-3 phosphorylation (Ser423/425) induced by TGF-β1. At 20 μM, TA completely blocked Smad-3 phosphorylation. Similarly, knocking down of HDAC6 by siRNA also resulted in a complete blockage of Smad3 phosphorylation in HPMCs exposed to TGF-β1 (Figure 3C–3D). Therefore, HDAC6 is a critical molecule in mediating the TGF-β1/Smad3 activation in peritoneal mesothelial cells undergoing EMT.

### Inhibition of HDAC6 suppresses phosphorylation of EGFR and STAT3 in peritoneal mesothelial cells

**Figure 4:**
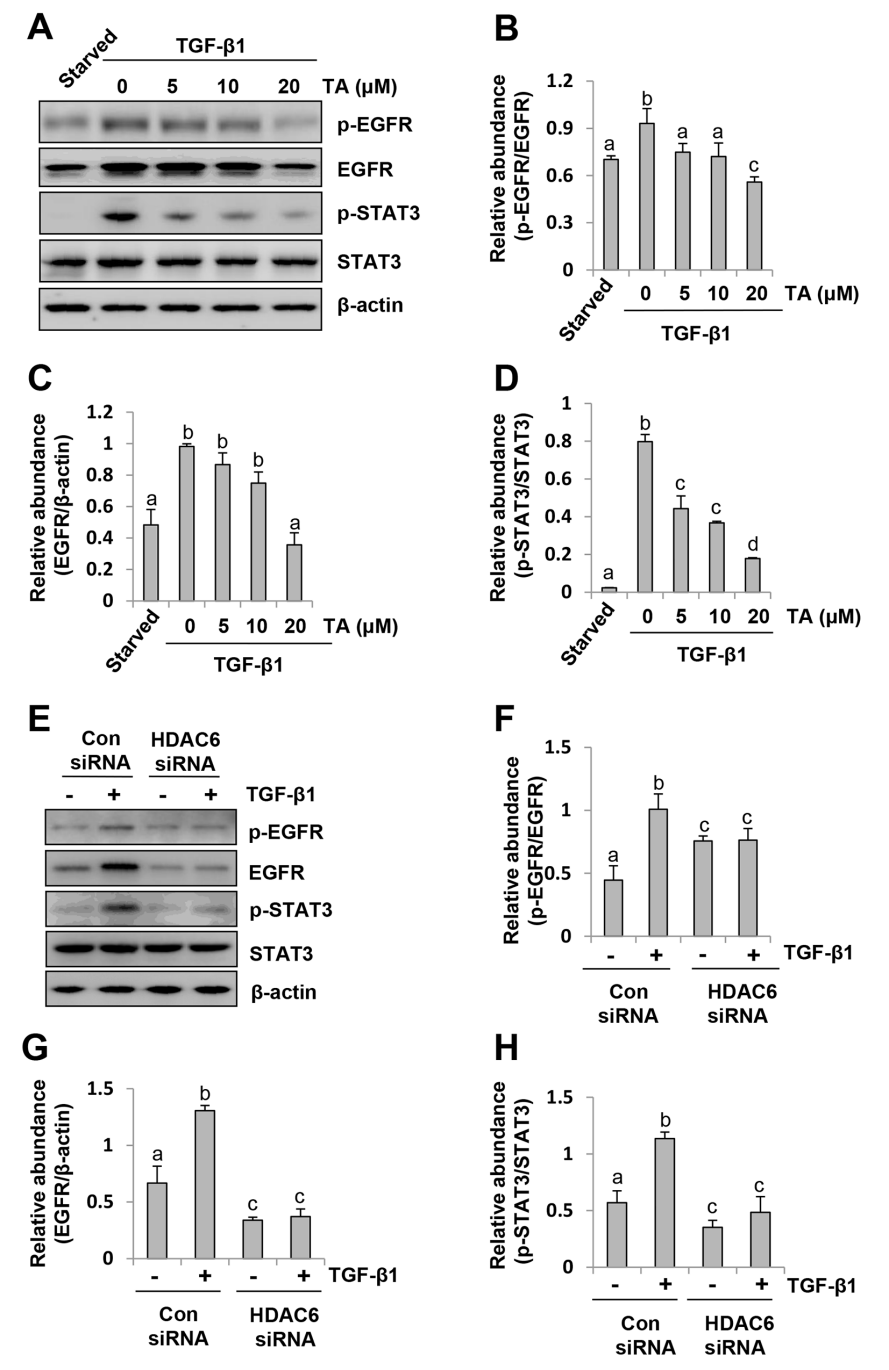
HDAC6 is required for phosphorylation of EGFR and STAT3 in peritoneal mesothelial cells exposed to TGF-β1 Serum-starved HPMCs were pretreated with various concentrations of TA (0-20 μM) for 1 hour **(A)** or transfected with siRNA targeting HDAC6 or scrambled (Con) siRNA for 24 hours **(E)** and then exposed to TGF-β1 (10 ng/ml) for an additional 24h. **(A, E)** Cell lysates were subjected to immunoblot analysis with antibodies p-EGFR, EGFR, p-STAT3, STAT3 or β-actin. **(B, F)** Expression level of p-EGFR was quantified by densitometry and normalized with EGFR. **(C, G)** Expression level of total EGFR was quantified by densitometry and normalized with β-actin. **(D, H)** Expression level of p-STAT3 was quantified by densitometry and normalized with total STAT3. Values are means±SD of at least three independent experiments. Bars with different letters (a-d) for each molecule are significantly different from one another (P<0.05).

Our recent studies have shown that activation of EGFR mediates the development of renal fibrosis and peritoneal fibrosis and that STAT3 acts downstream of EGFR [13, 35]. To demonstrate whether HDAC6 plays a role in regulating activation of EGFR during EMT, we examined the effect of TA and HDAC siRNA on the phosphorylation of EGFR and STAT3 (Tyr 705). As shown in Figure 4A-4D, exposure of HPMCs to TGF-β1 induced phosphorylation of EGFR and STAT3, and TA treatment inhibited their phosphorylation in a dose dependent fashion. A 20 μM dose of TA was sufficient to reduce their phosphorylation to the basal levels. Consistent with this result, knocking down of HDAC6 also completely blocked EGFR and STAT3 phosphorylation (Figure 4E, 4F, 4H). It should be noted that TGF-β1 induced expression of total EGFR but not total STAT3, and that blocking HDAC6 reduced expression of total EGFR, but not total STAT3 (Figure 4A, 4C, 4E, 4G). These data illustrated that HDAC6 also plays an important role in mediating activation/expression of EGFR and activation of STAT3.

### Administration of TA inhibits development of peritoneal fibrosis in a murine model of peritoneal fibrosis induced by high glucose dialysate

**Figure 5:**
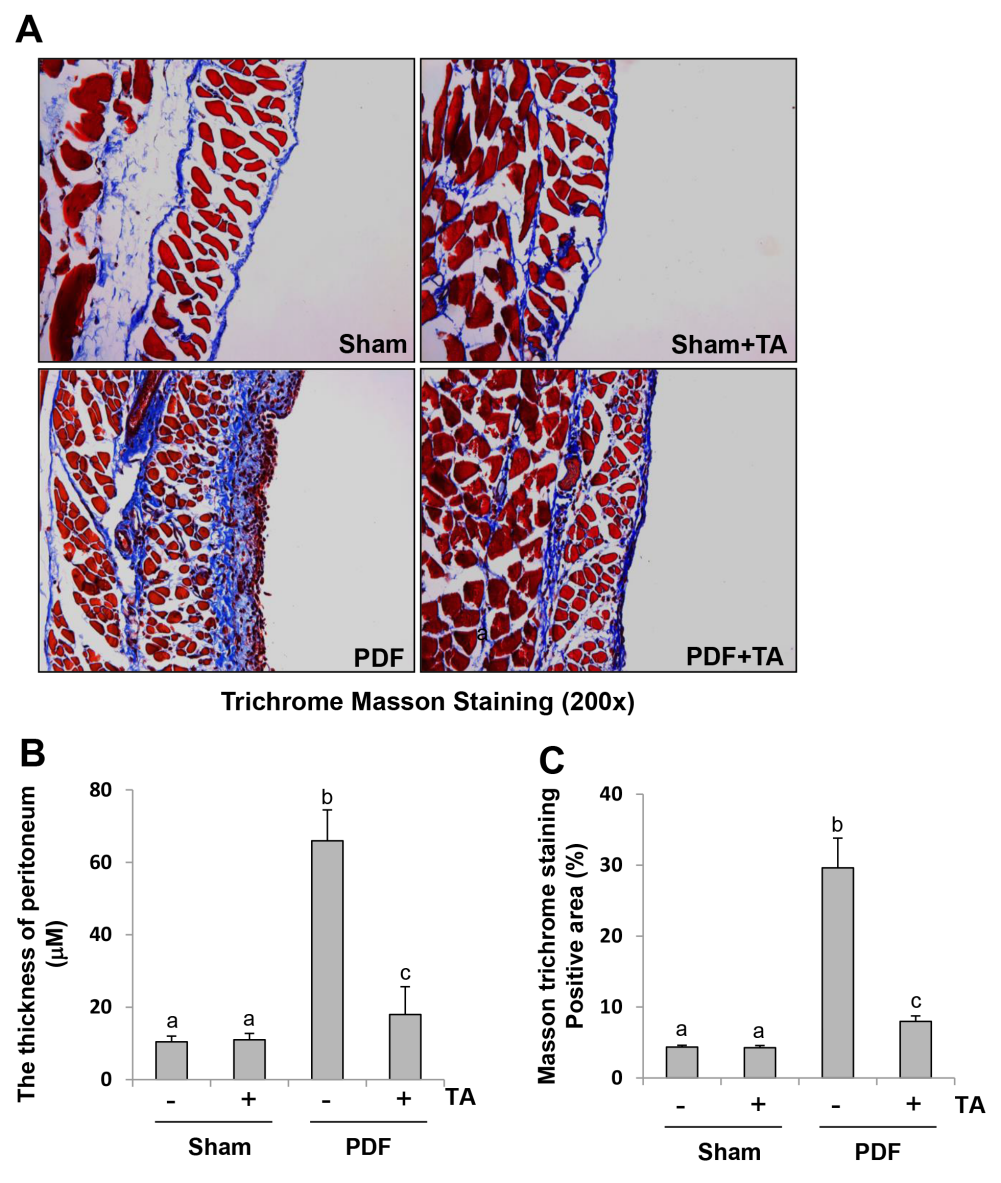
Administration of TA inhibits development of peritoneal fibrosis in a murine model of peritoneal fibrosis induced by high glucose dialysate Peritoneal membrane was collected at 28 days after PDF injection with or without administration of TA (70 mg/kg, daily). **(A)** Photomicrographs illustrate Masson trichrome staining of the peritoneum. **(B)** The graph shows the thickness of the compact zone measured from ten random fields (original magnification, ×200) of six mice peritoneal samples. **(C)** The graph shows the score of the Masson-positive submesothelial area (blue) from ten random fields (original magnification, ×200) of six mice peritoneal samples. Data are represented as the means±SD (n=6). Means with different letters (a-c) are significantly different from one another (P<0.05).

High glucose dialysate is a major factor leading to peritoneal fibrosis [36]. Based on the aforementioned role of HDAC6 in the EMT of cultured peritoneal mesothelial cells, we further evaluated the effect of HDAC6 inhibition on the development of peritoneal fibrosis. As shown in Figure 5, daily intraperitoneal injection of 3 ml 4.25% glucose dialysate in mice for 28 days successfully established a model of peritoneal fibrosis with features including the increased thickness of the submesothelial area, a layer of mature fibrous tissue containing collagen and elastin fibers as indicated by Masson trichrome staining. Administration of TA at 70 mg/kg every day immediately after injection of high glucose dialysate significantly reduced these pathological changes (Figure 5A–5C), suggesting that HDAC6 is a critical mediator of peritoneal fibrosis.

**Figure 6:**
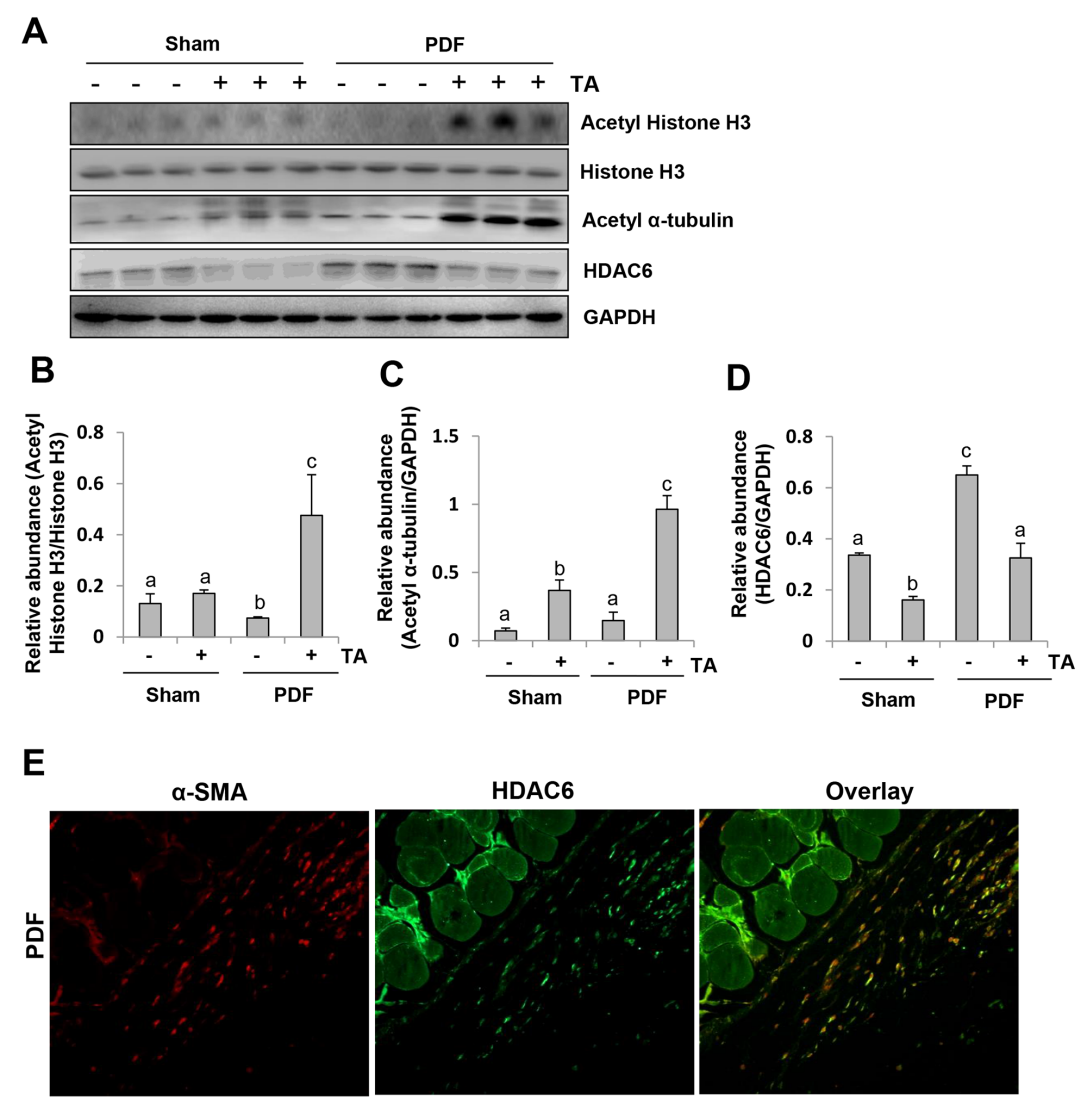
Inhibition of HDAC6 reduces histone H3 acetylation in the peritoneum of mice after exposure to high glucose dialysate Peritoneal membrane was collected at 28 days after PDF injection with or without administration of TA (70 mg/kg, daily). **(A)** The peritoneum was taken for immunoblot analysis of acetyl histone H3, histone H3, acetyl α-tubulin, HDAC6 or GAPDH; the representative results with three samples are shown. **(B)** Expression level of acetyl-histone H3 was quantified by densitometry and normalized with total histone H3. **(C and D)** Expression levels of acetyl α-tubulin (C) or HDAC6 (D) were quantified by densitometry and normalized with GAPDH. **(E)** Photomicrographs illustrate co-staining of α-SMA and HDAC6 in the peritoneum collected 28 days after 4.25%PDF injection (original magnification, ×200). Data are represented as the means±SD (n=6). Means with different letters (a-c) are significantly different from one another (P<0.05).

To demonstrate the effectiveness of TA *in vivo*, we examined the effect of TA on the expression of HDAC6, acetyl histone H3, and acetyl α-tubulin. As shown in Figure 6A-6D, an abundance of HDAC6 and basal levels of acetyl-histone H3 and acetyl α-tubulin were detected in the peritoneum of the sham group. Injection of 4.25% dialysate for 28 days resulted in a significant increase in the expression level of HDAC6, which was accompanied by decreased expression of acetyl histone H3 and acetyl α-tubulin. Administration of TA downregulated peritoneal HDAC6 to the basal level, while upregulating both acetyl histone H3 and acetyl α-tubulin in mice subjected to high glucose dialysate. Notably, TA treatment also reduced HDAC6 expression and slightly reduced the acetylation of histone H3 and α-tubulin in the peritoneum of control animals. Immunostaining showed that HDAC6 is mostly expressed in the cells present in the sub-mesothelial zone, co-localized with α-SMA, suggesting that they are myofibroblasts (Figure 6E). But few cells expressing HDAC6 alone were observed at the edge of the peritoneal membrane (Figure 6E). Collectively, these results illustrated that high glucose dialysate can induce HDAC6 expression in myofibroblasts and in the injured peritoneal mesothelial cells.

Collectively, these results illustrate that HDAC6 is involved in the development of peritoneal fibrosis.

### Inhibition of HDAC6 reduces fibroblast activation and ECM protein deposition in the peritoneum after exposure to high glucose dialysate

**Figure 7:**
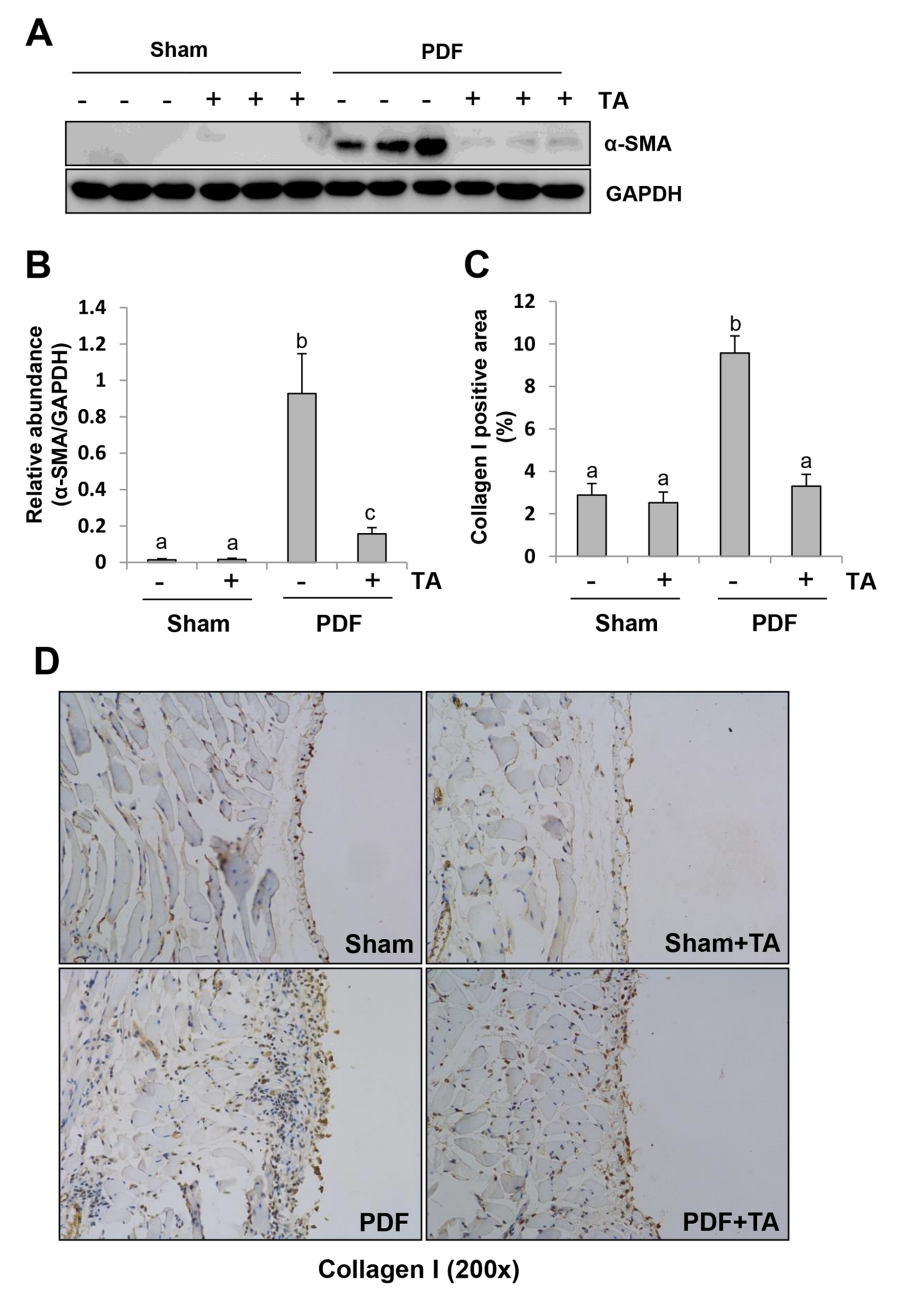
Inhibition of HDAC6 reduces fibroblast activation and ECM protein deposition in the peritoneum after exposure to high glucose dialysate Peritoneal membrane was collected at 28 days after PDF injection with or without administration of TA (70 mg/kg, daily). **(A)** The peritoneal tissue lysates were subjected to immunoblot analysis with specific antibodies against α-SMA or GAPDH. **(B)** Expression levels of α-SMA were quantified by densitometry and normalized with GAPDH. **(C)** The percentage of collagen I-positive areas was calculated from ten random fields of six mice peritoneal samples. **(D)** Photomicrographs illustrate immunohistochemical staining of collagen I in the submesothelial compact zone (original magnification, ×200). Data are represented as the means±SD (n=6). Means with different letters are significantly different from one another (P<0.05).

Peritoneal fibrosis is associated with the appearance of myofibroblasts and expansion of extracellular matrix [36]. To investigate the effect of TA on myofibroblast activation and extracellular matrix (ECM) deposition in the peritoneum after glucose exposure, we examined the expression of α-SMA by immunoblot analysis and collagen I expression by immunohistochemistry staining, respectively. As indicated in Figure 7A and B, a basal level of α-SMA was detectable in the peritoneum of mice, and its expression levels were dramatically increased after exposure to high glucose dialysate. TA treatment largely blocked α-SMA expression. Immunohistochemistry staining showed that expression of collagen I in the submesothelial compact zone was also significantly reduced after TA treatment (Figure 7C and 7D). These results, along with the data from Figure 5, suggest that pharmacological targeting of HDAC6 can prevent the development of peritoneal fibrosis.

### Inhibition of HDAC6 blocks activation of the TGF-β1/Smad signaling in the peritoneum exposed to high glucose dialysate

**Figure 8:**
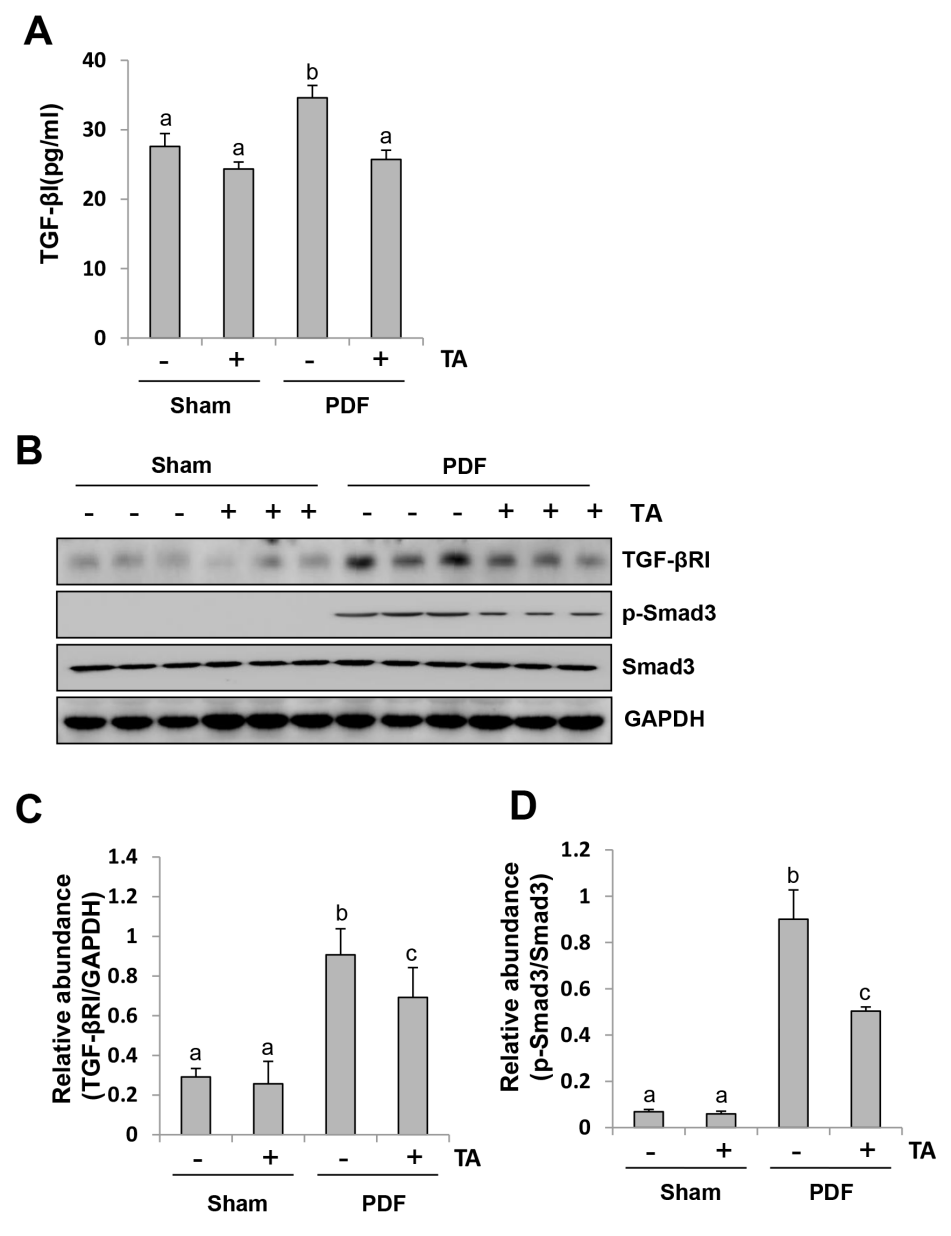
Inhibition of HDAC6 blocks activation of TGF-β1 signaling in the peritoneum induced by high glucose dialysate Peritoneal membrane was collected at 28 days after PDF injection with or without administration of TA (70 mg/kg, daily). **(A)** The peritoneal tissue lysates was used for measuring TGF-β1 by the ELISA. **(B)** The peritoneal tissue lysates were subjected to immunoblot analysis with specific antibodies against TGFβ-RI, p-Smad3, Smad3, or GAPDH. **(C)** Expression level of TGFβ-RI was quantified by densitometry and normalized with GAPDH. **(D)** Expression level of p-Smad3 was quantified by densitometry and normalized with total Smad3. Data are represented as the means±SD (n=6). Means with different letters are significantly different from one another (P<0.05).

The TGF-β1 signaling pathway plays a central role in regulating development of peritoneal fibrosis [15]. To demonstrate the role of HDAC6 in the activation of this signaling pathway during peritoneal fibrogenesis, we first examined the effect of TA on the expression of TGF-β1 in the peritoneum by ELISA. Figure 8A shows that peritoneum injured by high glucose PDF exhibited elevated levels of the TGF-β1, while TA treatment significantly reduced its expression. Next, we examined the effect of TA on the expression of transforming growth factor-beta receptor-I (TGF-βRI), phospho-Smad-3 and Smad-3 by western blot analysis. Figure 8B-8D demonstrated that TGF-βRI and Smad-3, but not phospho-Smad3, were expressed in the normal peritoneum. Injection of high glucose dialysate to the peritoneal cavity resulted in increased TGF-βRI expression and induced Smad-3 phosphorylation. TA treatment partially inhibited TGF-βRI expression and p-Smad-3 phosphorylation. These data suggest that HDAC6 is implicated in the activation of the TGF-β signaling pathway.

### Inhibition of HDAC6 abrogates the activation of EGFR/STAT3 signaling pathway in the peritoneum exposed to high glucose dialysate

**Figure 9:**
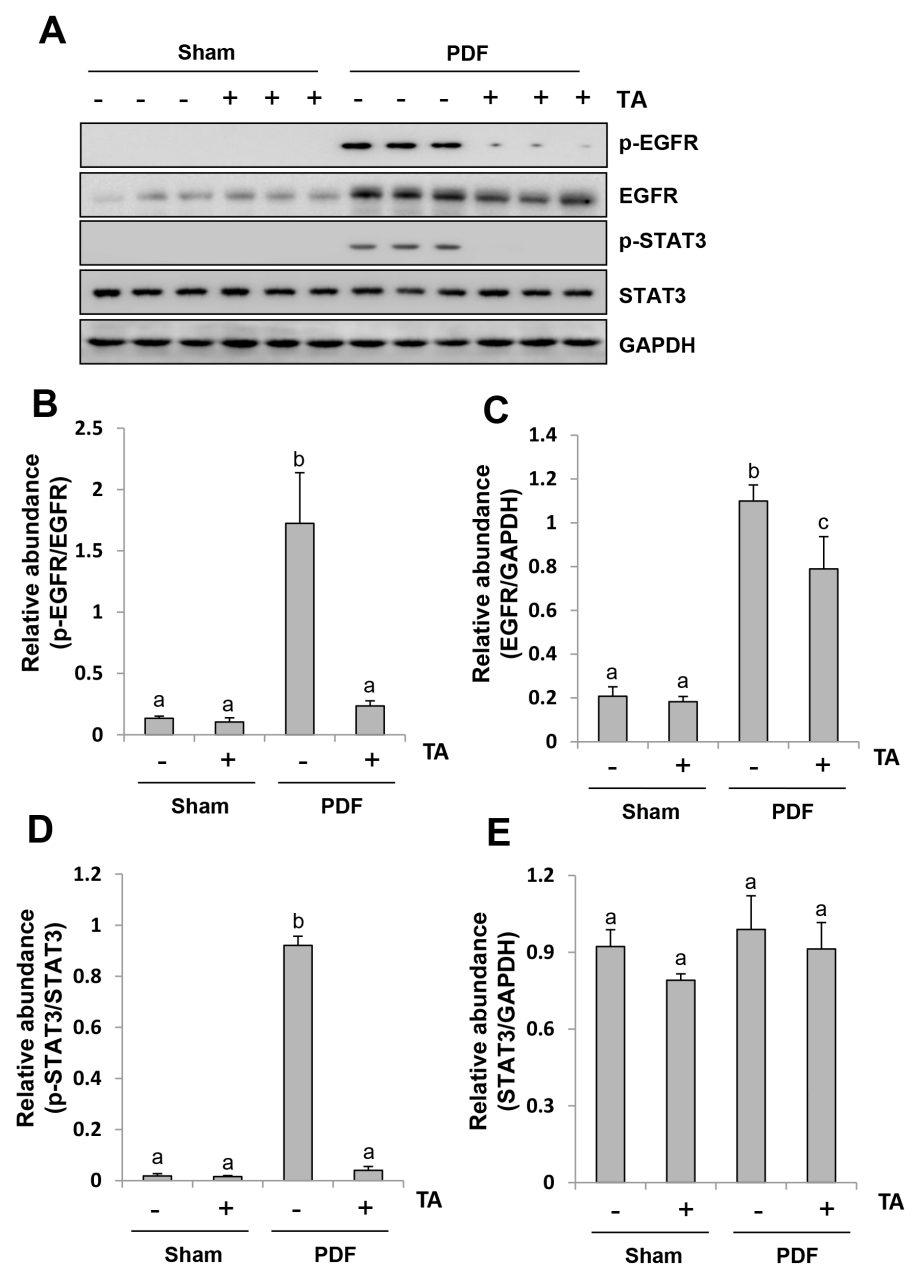
Inhibition of HDAC6 abrogates the activation of EGFR/STAT3 signaling pathway in the peritoneum exposed to high glucose dialysate Peritoneal membrane was collected at 28 days after PDF injection with or without administration of TA (70 mg/kg, daily). **(A)** The peritoneal tissue lysates were subjected to immunoblot analysis with specific antibodies against p-EGFR, EGFR, p-STAT3, STAT3 or GAPDH. **(B)** Expression level of p-EGFR was quantified by densitometry and normalized with total EGFR. **(D)** Expression level of p-STAT3 was quantified by densitometry and normalized with total STAT3. Expression levels of total EGFR **(C)** or total STAT3 **(E)** were quantified by densitometry and normalized with total GAPDH. Data are represented as the means±SD (n=6). Means with different letters are significantly different from one another (P<0.05).

Studies from our group and others have demonstrated that activation of the EGFR/STAT3 pathway is involved in the progression of peritoneal fibrosis [13, 37]. Thus, we set out to examine the effect of HDAC6 inhibition on the activation of EGFR and STAT3 (phosphorylation). As shown in Figure 9A, 9B, 9D, phosphorylated EGFR and STAT3 were significantly increased in the peritoneal membrane after high glucose dialysate exposure, while administration of TA resulted in a complete inhibition of EGFR and STAT3 phosphorylation. Interestingly, injection of high glucose dialysate increased expression of total EGFR, but not STAT3. TA treatment partially reduced expression of total EGFR (Figure 9A, 9C-9E). However, it seems that reduction of EGFR phosphorylation by TA treatment was not due to decreased expression of total EGFR. The ratio of p-EGFR/EGFR was still significantly lower in the peritoneal membrane treated with both high glucose dialysate and TA when compared with those treated with high glucose PDF alone (Figure 9B). These data indicate that inhibition of HDAC6 can also inactivate the EGFR/STAT3 signaling pathway and reduce EGFR expression levels in the injured peritoneal membrane.

### Inhibition of HDAC6 blocks activation of NF-κB signaling pathway and suppresses release of multiple inflammatory cytokines and chemokines

**Figure 10:**
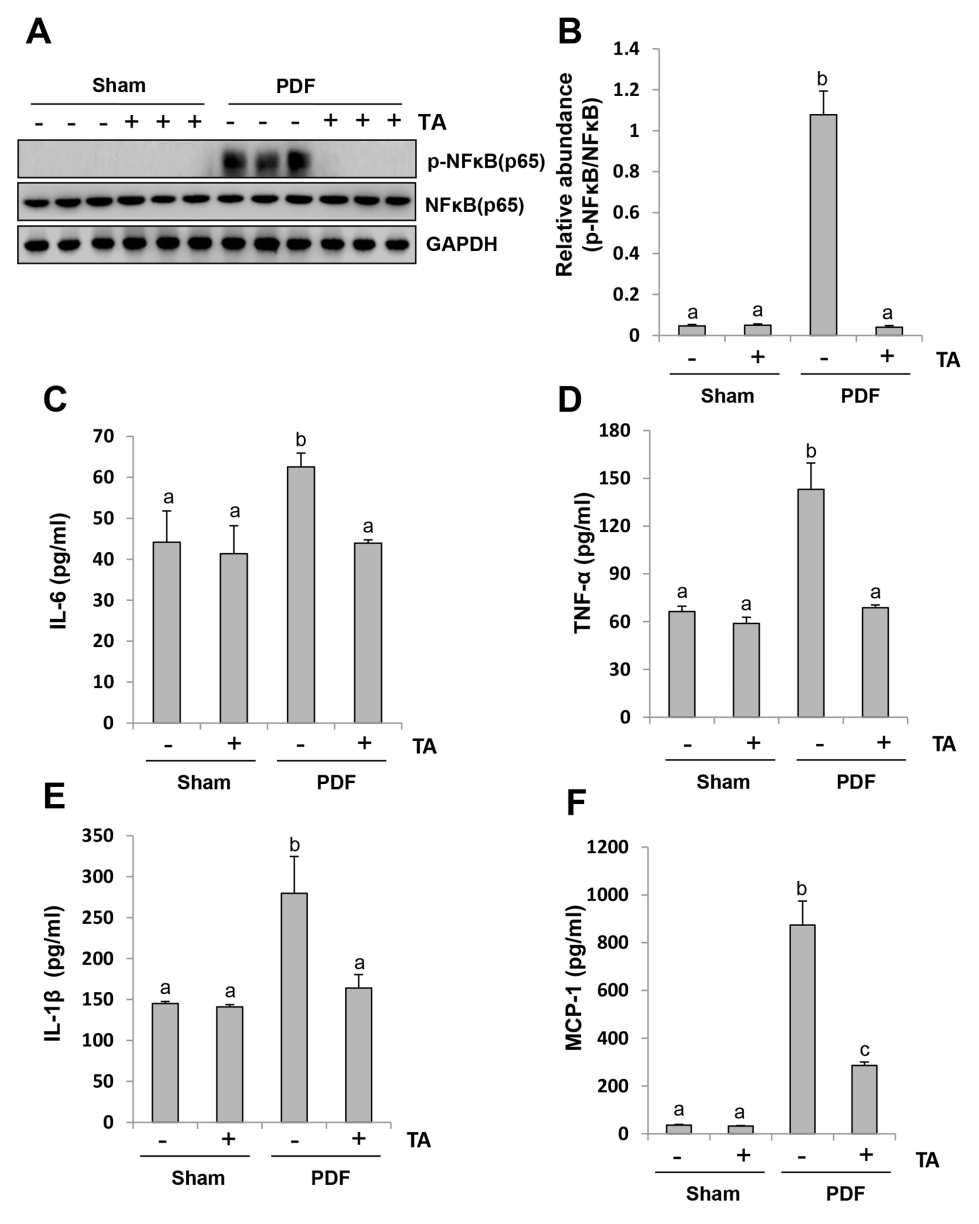
Inhibition of HDAC6 blocks activation of NF-κB signaling pathway and suppresses release of multiple inflammatory cytokines and chemokines Peritoneal membrane was collected at 28 days after PDF injection with or without administration of TA (70 mg/kg, daily). **(A)** The peritoneal tissue lysates were subjected to immunoblot analysis with specific antibodies against p-NF-κB, NF-κB or GAPDH. **(B)** Expression levels of p-NF-κB were quantified by densitometry and normalized with total NF-κB. Graphs show the expression levels of IL-6 **(C)**, TNF-α **(D)**, IL-1β **(E)**, and MCP-1 **(F)** by ELISA. Data are represented as the means±SD (n=6). Means with different letters are significantly different from one another (P<0.05).

NF-κB is a pivotal transcriptional factor involved in chemokine regulation, and its phosphorylation triggers the release of multiple inflammatory cytokines [38, 39]. As a result, we assessed the role of HDAC6 on the activation of NF-κB signaling and the subsequent production of inflammatory cytokines. As shown in Figure 10A and 10B, high glucose dialysate-induced injury of peritoneum led to a remarkable phosphorylation of NF-κB, which was completely suppressed by TA. The phosphorylated NF-κB was not detected in the peritoneum of the sham group with or without TA. Although an abundance of total NF-κB was expressed in the normal peritoneum, neither high glucose dialysate nor TA treatment altered its expression.

Since an abundant expression of inflammatory cytokines and chemokines is essential for the development and progression of peritoneal fibrosis, we further evaluated the effect of TA on the expression of some inflammatory cytokines and chemokines, including IL-6, IL-1β, TNF-α and MCP-1 by ELISA. As indicated in Figure 10, 10C-10F, all levels of these cytokine/chemokine were elevated in the peritoneum of mice administered with high glucose PDF and suppressed by TA treatment.

Taken together, these results indicate that HDAC6 activity is required for the activation of NF-κB signaling pathways and for the production and release of multiple inflammatory cytokines and chemokines in peritoneal fibrosis.

### Inhibition of HDAC6 attenuates macrophage infiltration in the peritoneum induced by high glucose PDF

**Figure 11:**
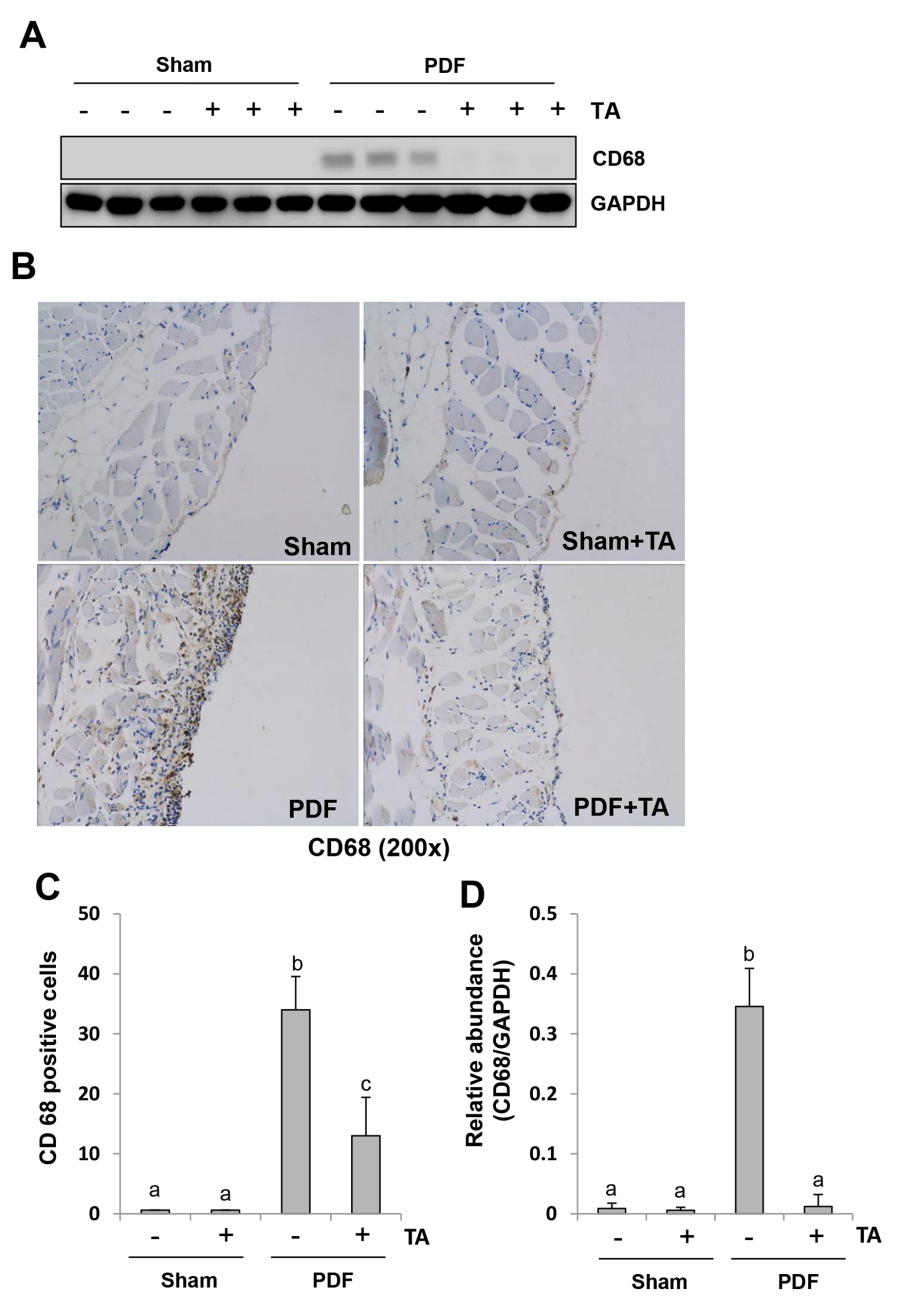
Inhibition of HDAC6 attenuates macrophage infiltration in the peritoneum induced by high glucose PDF Peritoneal membrane was collected at 28 days after PDF injection with or without administration of TA (70 mg/kg, daily). **(A)** The peritoneal tissue lysates were subjected to immunoblot analysis with specific antibodies against CD68 or GAPDH. **(B)** Photomicrographs (original magnification, ×200) illustrate CD68 staining of the peritoneal tissues. **(C)** The percentage of CD68-positive cells was calculated from ten random fields of six mice peritoneal samples. **(D)** Expression level of CD68 was quantified by densitometry and normalized with GAPDH. Data are represented as the means±SD (n=6). Means with different letters are significantly different from one another (P<0.05).

Increased macrophage infiltration in the submesothelial compact zone contributes to peritoneal fibrosis [13, 40]. To determine whether HDAC6 was involved in this response, we first assessed the level of CD68, a biomarker of macrophages. CD68 was not detectable in the normal peritoneum with or without TA treatment. Its expression levels were remarkably upregulated in the peritoneum following exposure to high glucose dialysate and downregulated to their basal level after TA treatment (Figure 11A and 11D). Next, we examined CD68 expression by immunohistochemistry and showed that the number of CD68 (+) macrophages was increased in the peritoneum after high glucose PDF injection and inhibition of HDAC6 also decreased their expressions (Figure 11B and 11C). Thus, these data suggest that HDAC6 activity is required for the infiltration of macrophages to the injured peritoneum.

### Inhibition of HDAC6 reduces angiogenesis and VEGF expression in the peritoneum after chronic exposure to high glucose dialysate

**Figure 12:**
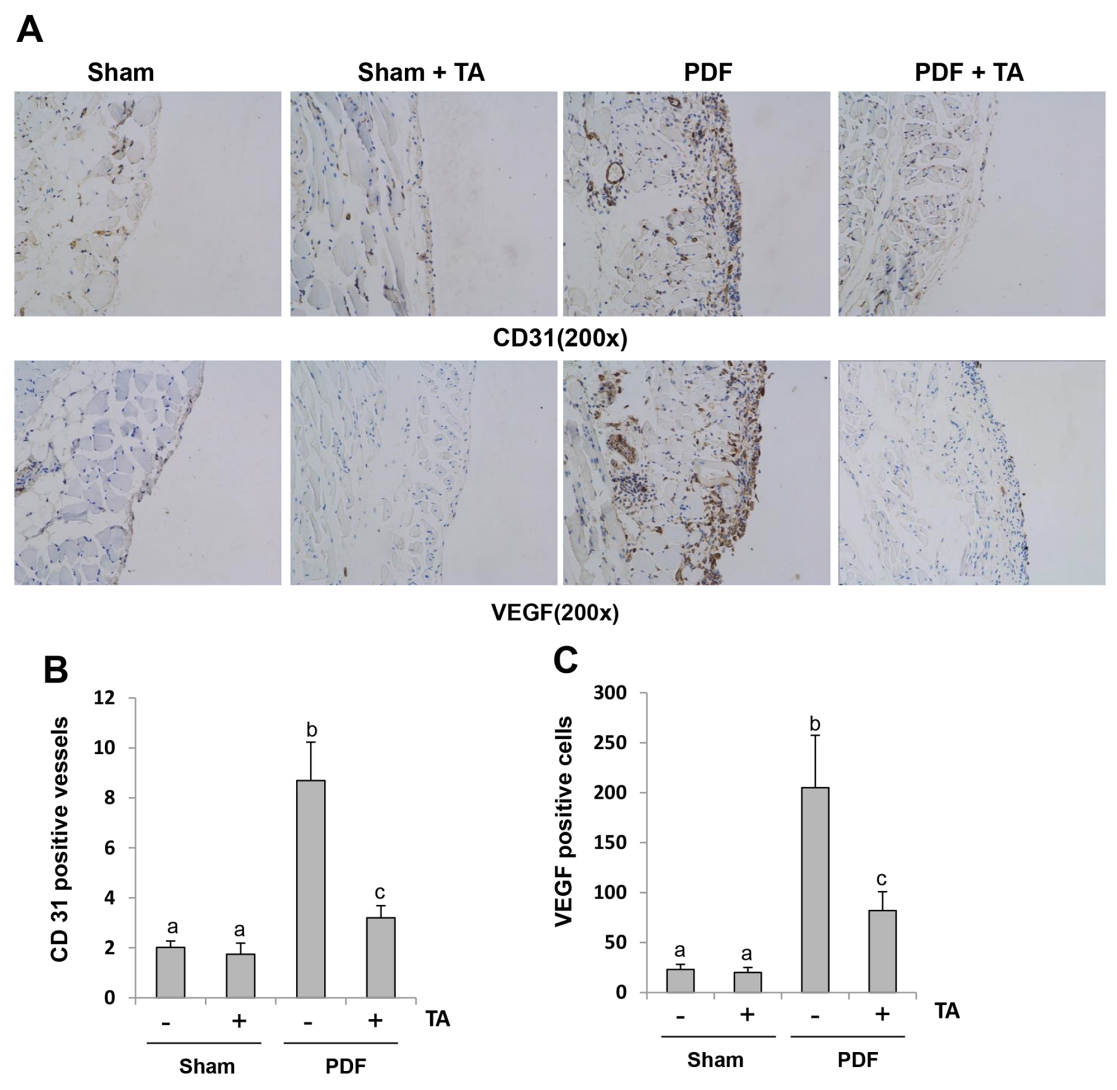
Inhibition of HDAC6 reduces angiogenesis and VEGF expression in the peritoneum after chronic exposure to high glucose dialysate Peritoneal membrane was collected at 28 days after PDF injection with or without administration of TA (70 mg/kg daily). **(A)** Photomicrographs (original magnification, ×200) illustrate CD31 staining and VEGF staining of the peritoneal tissues. **(B, C)** The percentage of CD31-positive vessels or VEGF-positive cells was calculated from ten random fields of six mice peritoneal samples. Data are represented as the means±SD (n=6). Means with different letters are significantly different from one another (P<0.05).

Angiogenesis is one of the pathologic consequences following long-term PD [4]. Previous studies suggested that VEGF expression is also implicated in peritoneal fibrosis [4, 41]. To investigate whether HDAC6 inhibition is involved in angiogenesis, we examined the expression level of CD31 (a marker of endothelial cells) and VEGF by immunohistochemistry. As shown in Figure 12, there are only a small number of CD31-positive vessels and VEGF-positive cells in the normal peritoneum, but their numbers were elevated in the injured peritoneum with high glucose dialysate. TA treatment significantly suppressed these responses. Therefore, these findings suggest that TA may also play a role in preventing the progression of peritoneal fibrosis through the inhibition of angiogenesis.

## DISCUSSION

Recent studies have demonstrated that HDAC6 inhibition can improve polycystic kidney disease [42], renal fibrosis [16] and acute kidney injury [33] in animal models. However, the role of HDAC6 in peritoneal fibrosis has not yet been reported. In this study, we demonstrated that inhibition of HDAC6 with TA (a highly selective inhibitor of HDAC6) or silencing with siRNA inhibits EMT in cultured human peritoneal mesothelial cells and attenuates activation of fibroblasts, deposition of ECM, induction of inflammatory responses, and angiogenesis in a mouse model of peritoneal fibrosis induced by chronic exposure to high glucose dialysate. These data demonstrate that HDAC6 is a critical mediator in peritoneal fibrosis and suggest that it may be a valuable therapeutic target for this complication.

Under normal conditions, mesothelial cells form a monolayer covering the peritoneal surface, displaying an epithelial-like appearance, while fibroblasts are embedded in the submesothelial interstitium [6]. Although our understanding of the mechanism of HDAC6 mediated peritoneal fibrosis remains incomplete, our results suggest that HDAC6 activation is critically involved in the EMT of peritoneal mesothelial cells and differentiation of fibroblasts into myofibroblasts. This is evidenced by our observations that pharmacological blockade or siRNA mediated silencing of HDAC6 inhibited TGF-β1-induced downregulation of E-cadherin (a marker of epithelial cells) and upregualtion of α-SMA and collagen I (hallmarks of mesenchymal like cells) in cultured mesothelial cells. In an animal model of peritoneal fibrosis induced by high glucose dialysate, we found that α-SMA is co-localized with HDAC6 in the cells in the submesothelial interstitium and administration of TA largely blocked expression of α-SMA. Although the source of myofibroblasts is still debated, inhibition of either EMT or the differentiation of local fibroblasts to myofibroblasts might attenuate development of renal fibrosis since both of mesothelial cells and myofibroblasts can produce collagen I, a major component of ECM [6] and we observed that TA treatment dramatically reduced collagen I expression in the peritonium. Mesothelial cells may contribute to peritoneal fibrosis through their capacity for producing EGF and CTGF. These growth factors are thought to target the mesothelium to induce the proliferation and myofibroblastic conversion of fibroblasts in a paracrine manner [43].

Our data suggest that HDAC6 inhibition prevents peritoneal fibrosis by blocking the TGF-β1/Smad signaling pathway. This is evidenced by our observations that blocking HDAC6 with TA significantly inhibited TGF-β1-induced phosphorylation of Smad-3 in cultured mesothelial cells and the expression of HDAC6-specific siRNA also impairs phosphorylation of Smad-3 by TGF-β1. *In vivo*, administration of TA was also able to inhibit TGF-β1 production, reduce TGF-βRI expression, block Smad-3 phosphorylation in injured peritoneal membranes. Currently, it remains unclear how HDAC6 activation promotes activation of the TGF-β1/Smad signaling. Previous studies suggest that binding of SMADs to microtubules keeps SMADs in their inactive stage, and TGF-β1 can trigger the release of SMADs from microtublules and the subsequent phosphorylation of SMADs [44]. As such, HDAC-dependent deacetylation of acetylated α-tubulin may alter microtubule functions and affect Smad phosphorylation. Nevertheless, the TGF-β1/Smad pathway may not be the sole pathway driving EMT or fibroblast activation. Peritoneal injury has been reported to be mediated by both Smad-3-dependent and Smad-3-independent mechanisms [45]. Further studies are necessary to determine the molecular mechanisms underlying HDAC6-mediated modulation of SMAD signaling.

HDAC6 inhibition may also attenuate peritoneal fibrosis via the targeting of EGFR signaling. Our recent studies have demonstrated that EGFR is a critical mediator of renal and peritoneal fibrosis [13]. In the current study, we found that inhibition of HDAC6 led to down-regulation and dephosphorylation of EGFR both in cultured mesothelial cells and in the injured peritoneum. Inhibition of HDAC activity also decreased the phosphorylation of STAT3, the downstream target of EGFR. In accordance with our observations, HDAC6 inhibition has been reported to promote EGFR degradation and normalize EGFR localization in Pkd1 mutant mouse embryonic kidney cells [46]. Since HDAC6 activation could result in increased expression of EGFR through disrupting microtubule mediated EGFR degradation via deacetylating α-tubulin in cystic epithelial cells, we further examined the effect of HDAC6 inhibition on α-tubulin acetylation in the injured peritoneal membrane. Our results showed that injury reduced α-tubulin acetylation whereas HDAC6 inhibition increased tubulin acetylation. These data suggest that inhibition of HDAC6 activity may reduce EGFR levels through inducing the degradation of EGFR.

HDAC6 inhibition-elicited suppression of inflammation may also contribute to attenuation of peritoneal fibrosis. The characteristic features of inflammation in peritoneal fibrosis are abundant expression of cytokines/chemokines and infiltration of macrophages [5]. In this study, we observed a significant elevation of multiple proinflammatory factors, including IL-6, TNF-α, IL-1β, and MCP-1, as well as macrophage infiltration of the peritoneum in mice injured by high glucose dialysate. Blockade of HDAC6 decreased expression of all these factors as well as infiltration by macrophages of the injured peritoneum. HDAC6 may control expression of these cytokines/chemokines by regulating key transcriptional factors. Here, we demonstrated that inhibition of HDAC6 with TA suppressed phosphorylation of NF-*κ*B (p65) and STAT3, two transcriptional factors vital for the expression and production of various inflammatory cytokines/chemokines [47].

Angiogenesis has been demonstrated as one of the key mechanisms of peritoneal fibrosis [48]. Increasing angiogenesis, one of the morphological alterations induced by PDF, ultimately results in ultrafiltration failure (UFF) [49]. *In vivo* and *in vitro* studies suggest that high density of vessels and progression of fibrosis are associated with an increase in TGF-β1 and VEGF expression [48]. Moreover, clinical experiments also find a tight connection between density of vessels and fibrosis in patients receiving PD treatment [50]. On this basis, we speculated that HDAC6 may also play a role in regulating this process. We indeed observed that HDAC6 inhibition reduced the expressions of CD33, a marker for the density of vessels as well as VEGF, a key growth factor for promoting blood vessel growth in the PDF-induced fibrotic peritoneum. Thus, inhibition of angiogenesis may be a significant mechanism associated with the anti-fibrotic effects of HDAC6 inhibitors.

HDAC6 inhibition has recently emerged as an attractive target for the treatment of cancer and other chronic diseases. Numerous studies have shown that HDAC inhibitors are able to prevent cell migration, apoptosis, angiogenesis and DNA repair [51–53]. There are also studies demonstrating the effectivity of HDAC6 inhibitors in treating chronic diseases such as neurodegenerative diseases, cardiovascular diseases, and cystogenesis [29]. Our studies suggest that TA, a special inhibitor of HDAC6, can alleviate the development and progression of peritoneal fibrosis, which provides a rationale for using HDAC6-specific inhibitors as potential anti-fibrotic drugs in clinical trials. So far, four HDAC inhibitors, vorinostat (or SAHA), romidepsin, belinostat and panobinostat have been approved for the treatment of haematological malignancies [29], but their application in chronic diseases may be limited by their adverse effects, in particular, haematological toxicity and QT prolongation [29]. Since HDAC6 KO mice do not show abnormal function of major organs [31], there may promise in developing HDAC6 inhibitors as drugs for use in the prevention and/or treatment of chronic complications like peritoneal fibrosis. Currently, two isoform-specific HDAC6 inhibitors, ricolinostat (ACY-1215) and ACY-241 are under phase I/II clinical evaluation for the treatment of patients with multiple myeloma and lymphoid malignancies [29]. Our results suggest that a clinical trial aimed at preventing and/or treating peritoneal fibrosis by using HDAC6 inhibitors might be possible.

**Figure 13:**
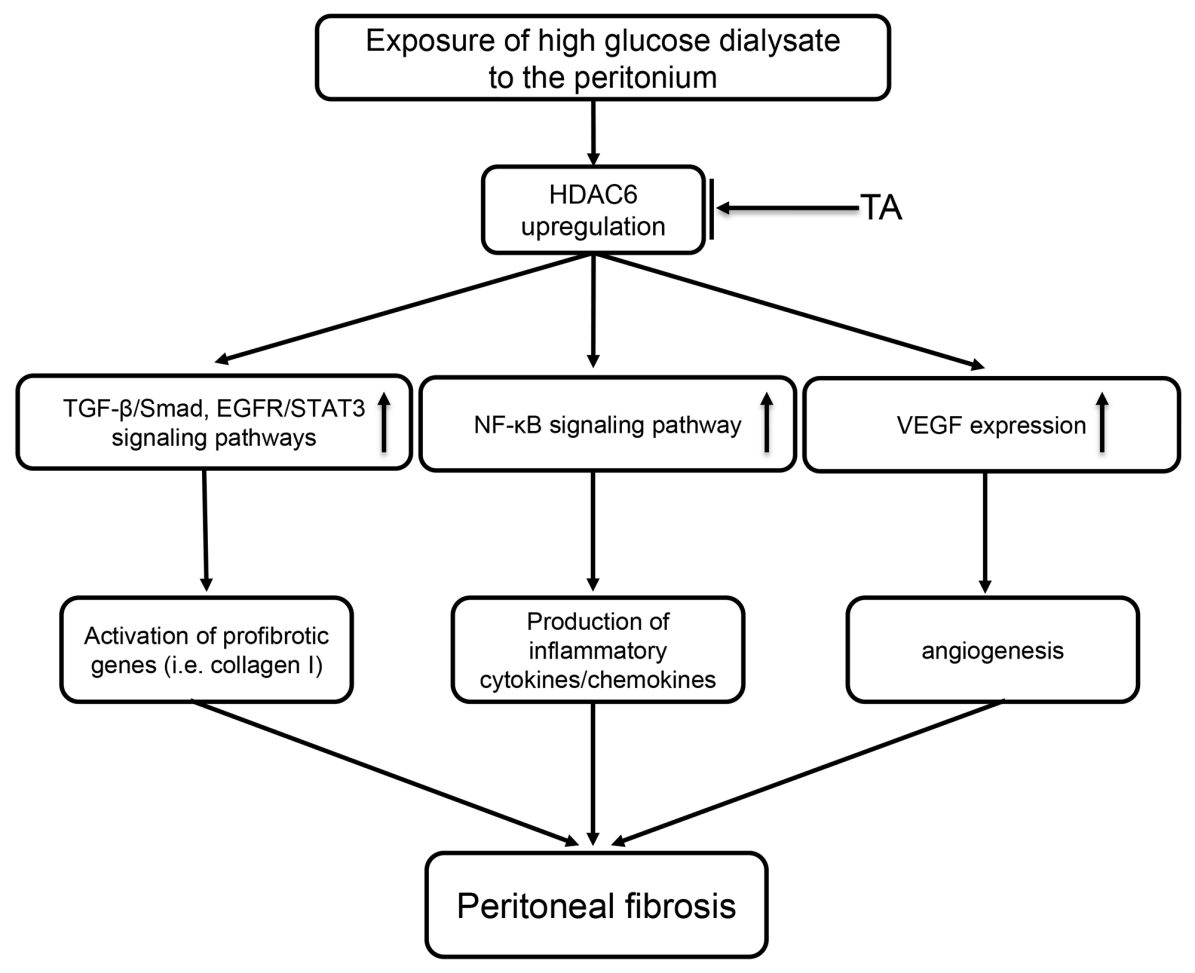
Signaling pathways of HDAC6 inhibition-elicited attenuation of peritoneal fibrosis Exposure of high glucose dialysate to the peritonium upregulates HDAC6, which subsequently leads to activation of profibrotic signaling pathways (i.e. TGFβ/Smad, EGFR/STAT3), induction of proinflammatory responses (i.e. activation of NF-κB, production of multiple cytokines/chemokines) and triggering angiogenesis via increasing VEGF production. All these responses are inhibited by TA, a highly selective HDAC6 inhibitor.

In conclusion, we demonstrated for the first time that the inhibition of HDAC6 is successful in preventing the development of peritoneal fibrosis. These anti-fibrotic effects may be associated with inactivation of the TGF-β1/Smad3 and EGFR/STAT3 signaling pathways, inhibition of NF-κB activation and inflammatory responses, and reduction of VEGF production and subsequent angiogenesis (Figure 13). Thus, HDAC6 may represent a viable target for the prevention and treatment of peritoneal fibrosis.

## MATERIALS AND METHODS

### Chemicals and antibodies

Tubastatin A was purchased from Selleckchem (Houston, TX, USA). Antibodies to p-STAT3 (Tyr-705), STAT3, p-Smad3 (Ser423/425), Smad3, p-EGFR (Tyr1068), p-NF-*κ*B (Ser536), NF-*κ*B, Histone H3, Acetyl Histone H3 (Lys9), Acetyl α-tubulin (Lys40) and HDAC6 were purchased from Cell Signaling Technologies (Danvers, MA, USA). Antibodies to collagen I (A2), E-cadherin, TGF-βRI, CD68, EGFR, VEGF, CD31 and GAPDH (glyceraldehyde 3-phosphate dehydrogenase) as well as HDAC6 siRNA were purchased from Santa Cruz Biotechnology (San Diego, CA, USA). Antibody to β-actin was purchased from TransGen Biotech (Beijing, China). TNF-*α*, IL-1β, TGF-β1, MCP-1, IL-6 and TGF-β1 enzyme-linked immunosorbent assay (ELISA) kits were from R&D Systems (Minneapolis, MN, USA). α-SMA, DMSO and other chemicals were obtained from Sigma-Aldrich (St. Louis, MO, USA).

### Cell culture and treatments

HPMCs were cultured in DMEM/High glucose (Sigma-Aldrich) containing 10% FBS, 1% penicillin and streptomycin in an atmosphere of 5% CO_2_, and 95% air at 37°C. To determine the effect of HDAC6 inhibition on fibrosis induced by TGF-β1, HPMCs were starved for 24 hours with 0.5% FBS DMEM and then exposed to TGF-β1(10 ng/ml) for 24 hours in the presence or absence of different doses of TA before cell harvesting.

### siRNA transfection

The small interfering (si) RNA oligonucleotides targeted specially for HDAC6 were used in this study. HPMCs were seeded to 30-40% confluence in antibiotic-free medium and grown for 24 hours, and then were transfected with HDAC6 siRNA (50 nmol) with lipofectamine 2000. In parallel, scrambled siRNA (50 nmol) was used as control for off-target changes in HPMCs. At 24h after transfection, cells were treated with TGF-β1 (10 ng/ml) for an additional 24h before being harvested for the experiments.

### Peritoneal fibrosis mice model and TA treatment

The peritoneal fibrosis model was established in C57/BL6 mice weighing 20-25 g (Shanghai Super-B&K Laboratory Animal Corp. Ltd.). Peritoneal fibrosis in mice was created by intraperitoneal injection of 3 ml peritoneal dialysis solution with 4.25% glucose (Baxter) every day for 28 days [1]. To investigate the effect of HDAC6 inhibition on peritoneal fibrosis, 70 mg/kg TA was delivered by daily intraperitoneal injection in DMSO vehicle. Mice were randomly divided into four groups with 6 mice per group: the sham group with DMSO, sham administered TA group, the peritoneal fibrosis group and mice with peritoneal fibrosis administered TA. At the end of 28 days, all mice were sacrificed and the parietal peritoneum was collected for further analysis. All the experiments were conducted in accordance with the animal experimentation guideline of Tongji University School of Medicine, China.

### Immunoblot analysis

Immunoblot analysis of peritoneum tissue samples was conducted as described previously [14]. The densitometry analysis of immunoblot results was conducted by using ImageJ software (National Institutes of Health, Bethesda, MD, USA).

### Morphologic studies of peritoneum

Formalin-fixed peritoneum were embedded in paraffin and prepared in 3-*μ*m-thick sections. For evaluation of peritoneal fibrosis, Masson trichrome staining was performed according to the protocol provided by the manufacturer (Sigma-Aldrich). The thickness of the submesothelial tissue was evaluated (in micrometers), and the average of ten independent measurements was calculated for each section (original magnification, ×200).

### Immunohistochemical staining

Immunohistochemical staining was conducted on the basis of the procedure described in our previous studies [14]. For quantitative assessment, the positive staining area was measured by ImageJ software, and the average ratio to each microscopic field (original magnification, ×200) was calculated and graphed. Immuofluorescent staining was carried out according to the procedure described in our previous studies [35]. Images were taken using a Zeiss 710 Duo microscope.

### ELISA analysis

ELISA detection of TGF-β1, MCP-1, IL-1β, TNF-*α*, IL-6 protein was performed in accordance with the manufacturer’s instructions.

### Statistical analysis

All the experiments were conducted at least three times. Data depicted in graphs represent the means ± SD. for each group. Intergroup comparison was made using one-way analysis of variance. Multiple means were compared using Turkey’s test. The differences between two groups were determined by Student’s *t* test. Statistical significant difference between mean values was marked in each graph. *P<*0.05 is considered significant. The statistical analyses were conducted by using IBM SPSS Statistics 20.0 (Beijing, China).
